# A Rare Case of Primary Chondrosarcoma of the Breast: A Case Report and Comprehensive Literature Review

**DOI:** 10.15190/d.2024.12

**Published:** 2024-09-30

**Authors:** Sujata Agrawal, Zachariah Chowdhury, Paramita Paul, Tanima Mandal

**Affiliations:** ^1^Department of Oncopathology, Homi Bhabha Cancer Hospital (HBCH) and Mahamana Pandit Madan Mohan Malviya Cancer Centre (MPMMCC), Tata Memorial Centre, Homi Bhabha National Institute (HBNI), Varanasi, India

**Keywords:** Breast chondrosarcoma, breast sarcoma, chondromatous lesion, immunohistochemistry

## Abstract

Breast sarcomas are a diverse group of malignant neoplasms originating from the mammary stroma. They are uncommon tumors, often occurring as a component of other tumors. Among malignant breast mesenchymal tumors, pure sarcomas lacking epithelial components are even rarer, comprising only 0.5% of breast tumors. The most common types include angiosarcomas, liposarcomas, and osteosarcomas. Pure, primary, and de novo chondrosarcomas are exceedingly rare within breast sarcomas, with very few cases reported. Distinguishing them from metaplastic carcinoma and phyllodes tumors with chondromatous areas entails extensive sampling to exclude proliferating ductal elements. Herein, we present a case of primary chondrosarcoma of the breast in a 72-year-old Indian woman. Initial core biopsy suggested a primary chondroid neoplasm or a heterologous component of a phyllodes tumor. The patient underwent modified radical mastectomy, and histological examination confirmed chondrosarcoma, grade 1, after thorough sampling.Clinical Relevance: This case emphasizes the necessity of incorporating rare sarcomatous breast tumors into the differential diagnosis for breast masses, especially those with chondroid differentiation. The report also reinforces the pivotal role of accurate histopathological evaluation in guiding appropriate surgical and adjunctive treatment, which can significantly impact prognosis in such rare malignancies.

## INTRODUCTION

Pure breast sarcoma is an extremely rare and heterogeneous group of malignancies, constituting less than 1% of total breast malignancies and less than 5% of all soft tissue sarcomas (STS)^1^. They arise from the breast connective tissue cells and account for approximately 4.6 new cases per million women per year^2^. Due to its rarity, published literature is limited and confined to small retrospective case reviews and case reports. It is crucial to recognize them as a distinct entity and discern them from the more common breast carcinomas so that the differences in their behaviour can be accounted for when planning therapy. Particularly, when chondrosarcomatous areas are evident on histopathological analysis, these tumors should be considered in the differential diagnosis of breast tumors.

## CASE HISTORY

**Figure 1:**
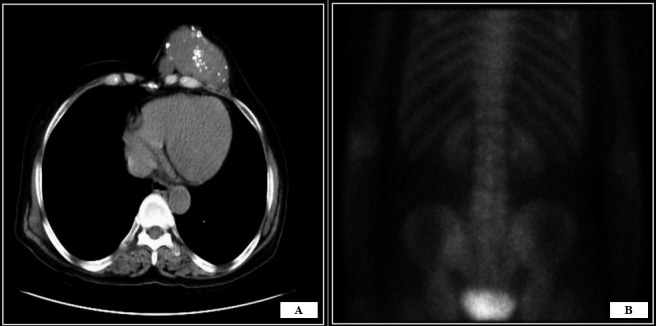
Figure 1. Imaging Findings of Breast Chondrosarcoma (A). CT thorax shows an ill-defined, minimally heterogeneously enhancing mass involving the upper inner quadrant of the left breast, measuring approximately 8.3 x 5.7 cm. It involves the underlying chest wall muscles. However, underlying rib is unremarkable, and (B). 99m-TC-MDP Bone Scan shows no scintigraphic evidence of skeletal metastases.

A 72-year-old female presented with a lump in her breast for the last 1 year, with notable accelerated growth in the past month. She had no medical history and no family history of breast cancer. Physical examination revealed a painless palpable hard mass measuring 9x8 cm in the central quadrant of the left breast, fixed to the chest wall with no evident axillary lymphadenopathy. Her contralateral breast and axilla were normal on clinical examination. Computed Tomography (CT) scan revealed an ill-defined, minimally heterogeneously enhancing mass measuring approximately 8.3 x 5.7 cm, posteriorly involving the underlying chest wall muscles without rib involvement (Figure 1A). Areas of suspicious microcalcifications were seen scattered within the mass. Subcentimeter-sized reactive nodes were seen in the left axillary region. The CT scan impression was suspicious for a phyllodes tumor. Biopsy was performed from the breast mass, which unveiled a chondroid lesion composed of lobules of atypical chondrocytes in a chondromyxoid background. Cellularity was mildly increased. No mitoses or necrosis were noted. No breast parenchyma was identified. Histopathological impressions were either a primary chondroid neoplasm or a heterologous component of a phyllodes tumor. A 99M-TC-MDP [Technetium 99m Methylene Diphosphonate] bone scan did not evince any skeletal involvement or metastases (Figure 1B).

The patient underwent a left modified radical mastectomy (MRM) with nodal axillary clearance of levels I and II.

## PATHOLOGIC FINDING

**Figure 2:**
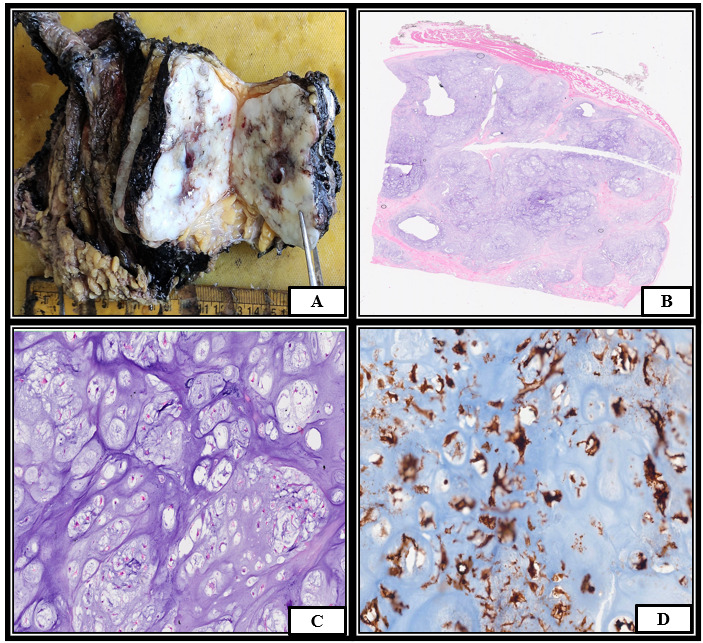
Figure 2. Pathological Findings of Mastectomy Specimen (A). Left mastectomy specimen shows a solid, lobulated, firm, pearly white tumor with focal degeneration. (B). Photomicrograph of the histopathology: scanner view shows a well-circumscribed lesion involving the chest wall muscles (H&E, 2x). (C). High-powered (H&E, 20x) view demonstrating a cartilaginous tumor with interspersed nodular and lobulated islands of well-differentiated cartilage. Lacunar spaces are occupied by pleomorphic chondrocytes with hyperchromatic nuclei, some showing binucleation. (D). Chondrocytes show immunoreactivity for S-100 on immunohistochemistry (20x).

On gross examination, the left MRM specimen measured 19x12.5x6 cm, and on cut section revealed a solid, lobulated, firm, pearly white tumor measuring 7.5x7.5x5.5 cm (Figure 2A). Focal areas of degeneration and haemorrhage were also noted. Microscopic analysis demonstrated a well-circumscribed tumor composed of lobules of hyaline cartilage with focal haemorrhage and necrosis (Figure 2B). Cellularity was slightly increased with the lacunar spaces occupied by pleomorphic chondrocytes with hyperchromatic nuclei, some showing binucleation. Mitosis was infrequent. There was no evidence of stromal overgrowth, epithelial hyperplasia, ductal carcinoma in situ or malignancy, or any other mesenchymal element (Figure 2C). Adjacent breast parenchyma disclosed few compressed terminals duct lobular units. The entire specimen was extensively sampled multiple times mainly in the search for epithelial proliferative components, to rule out phyllodes tumor with heterologous component or metaplastic breast carcinoma (MBC). Immunohistochemical studies revealed S100 positivity in the atypical chondrocytes (Figure 2D). The final diagnosis proffered was atypical cartilaginous tumor/chondrosarcoma Grade 1. The patient was discharged in a fine condition postoperatively with plan for adjuvant radiotherapy but was lost to follow-up.

DISCUSSION

Primary breast sarcomas are highly rare; they represent a heterogeneous group of tumors, with a prevalence of < 1% of all breast tumors^1^, and in particular primary breast chondrosarcomas are extremely uncommon with very few cases reported in the published literature. As a primary breast tumor, chondrosarcoma might occur in three different forms: as a pure neoplasm (pure chondrosarcoma), as the stromal component of a histologically malignant phyllodes tumor, or as chondrosarcomatous differentiation in a MBC. The one we chanced upon was a case of histologically pure primary chondrosarcoma. Primary chondrosarcoma of the breast is an extremely rare entity typically occurring in women over 40 years old and are generally large but do not commonly invade the skin or regional lymph nodes^3-7^. The current case aligns with the clinical findings reported in previous studies.

Before diagnosing primary chondrosarcoma of the breast, it is essential to exclude other potential differential diagnoses. Excluding malignant phyllodes tumors with predominant chondrosarcomatoid components can be particularly challenging. Malignant phyllodes tumors often exhibit a leaf-like architecture with stromal overgrowth and a biphasic pattern of epithelial and stromal components. The stromal component commonly manifests atypical features, such as increased cellularity and mitotic figures, and may display areas resembling chondrosarcoma. Extensive sampling is mandatory in the quest for proliferating epithelial components, as was performed in our case, to preclude phyllodes and settle for a diagnosis of pure sarcoma. Thus, not only in a scenario associated with chondrosarcoma but also with any other pure mesenchymal neoplasm, the aforementioned strategy is pivotal for veracious recognition in such contexts.

MBC with heterogeneous mesenchymal differentiation is another entity bracketed in such a diagnostic quandary, for which the previously alluded grossing manoeuvre is indispensable. The epithelial component for MBC must be proliferating and malignant, and there may be no direct transition between the carcinomatous and sarcomatous components. In a morphologically equivocal setting where the epithelial element is not overtly manifest, a diagnosis of MBC can be plausible based on the evidence of epithelial differentiation by immunohistochemical analysis^8^. To diagnose primary chondrosarcoma of the breast, adjacent non-mammary site of origin, especially the rib, needs to be excluded clinically and radiologically. Chondrosarcoma of the rib often shows a mass arising from the rib and extending into the breast tissue, which can be better delineated on imaging modalities such as CT or Magnetic Resonance Imaging. Bone involvement, such as rib destruction or remodelling, is usually evident^9^. However, primary chondrosarcoma of the breast typically appears as a well-defined mass within the breast tissue. Imaging may show calcifications within the tumor, but the tumor will be confined to the breast. Histologically, primary chondrosarcoma of the breast is characterized by malignant cartilage cells (chondrocytes) within lacunae and the absence of epithelial components. In contrast, chondrosarcoma of the rib shows the presence of bone tissue or direct continuity with rib structures.

**Table 1:** Review of the cases of primary breast chondrosarcomas reported in the literature ND: Not done, NA: Not available, DOD: Died of disease, USG: Ultrasonophrapy, FNAC: Fine needle aspiration cytology, ER: Estrogen receptor, PR: Progesterone receptor, NEM: No evidence of malignancy, RT: Radiotherapy, and MRI: Magnetic resonance imaging.

First author, year (reference)	Gender (M/F)	Age, (years)	Later ality	Size of tumor (cms)	Lymph node Status	Hormonal status	Therapy	Follow-up
Beltaos E et al. 1979 ^10^	F	73	Left	25x2015	ND	ND	Simple mastectomy	NA
	F	51	Left	5.5x5x 5	Neg	ND	Radical mastectomy	Lung metastasis, DOD
Gupta et al. 2003 ^3^	F	46	Left	15x14	Neg	ER/PR/ Her2neu:Neg	Mastectomy and axillarylymphadenectomy	1 year, NEM
Verfaille et al. 2005 ^11^	F	77	Right	3	Neg	ND	Mastectomy with sentinel nodeexcision	1 year, NEM
Gurleyik et al. 2009 ^4^	F	52	Right	5x5x3	Neg	ER/PR/ Her2neu: Neg	Mastectomy and axillary lymphadenectomy	NA
Lakshmikant et al.2010 ^5^	F	42	Left	13x10x 6	Neg	ND	Mastectomy andaxillary lymphadenectomy	NA
Patterson et al. 2012 ^6^	F	52	Left	5.6x4.1 x2.8	Neg	ND	Mastectomy and RT	1 year, NEM
Badyal et al. 2012 ^7^	M	80	Right	20x10	Neg	ND	Mastectomy with axillary lymphadenectomy and RT	YES
Mujtaba et al. 2013 ^12^	F	40	Right	21 x 19x 11	ND	ND	Mastectomy with RT	NA
Errarhay et al. 2013 ^13^	F	24	Right	1-1.5	ND	ER/PR/ Her2neu:Neg	Mastectomy	NA
Bagri et al.2015^14^	M	65	Right	10.4x10.3x9.9	ND	ER/PR: Neg	Mastectomy	Lost to follow-up after 3 months
Pasta V et al 2015 ^15^	F	63	Right	6.5×4.5×5	ND	ND	Wide quadrantectomy	2.5 years, NEM
Amadu AM et al 2017^16^	F	62	Right	9x9	ND	ER/PR/ Her2neu:Neg	Mastectomy	NA
Current case	F	72	Left	7.5x7.5 x5.5	Neg	ND	Modified radical mastectomy with axillary clearance (I-II)	3months, NEM

Estrogen receptor (ER), progesterone receptor (PR), and Her2/neu receptor status testing were conducted in a few cases, and all documented ER/PR/Her2/neu results were negative. Appraisal of ER/PR receptors was not executed in our case, since only atypical chondrocytes were observed, and therefore, estrogen antagonists were not administered to the patient. Following the standard protocol for other sarcomas treated at our facility, the patient was referred for adjuvant therapy.

Treatment of chondrosarcoma of the breast focuses primarily on complete surgical resection with clear margins, often supplemented by reconstructive surgery. Multimodality treatment may decrease local and systemic recurrence rates of somatic sarcomas, but results are inconclusive in patients with breast sarcomas. The roles of radiation and chemotherapy are limited and are considered on a case-by-case basis. Regular follow-up and a multidisciplinary approach are crucial in managing this rare malignancy effectively. The prognosis of chondrosarcomatous breast tumors remains unclear due to rarity of published data and a lack of comprehensive clinical or morphologic information in many reported cases, posing challenges for analysis (Table 1).

## CONCLUSION

Primary chondrosarcoma of the breast is an extremely rare tumor and thus can be fraught with misdiagnosis. It warrants cogitation when chondroid elements are discovered on diagnostic modalities and apposite pathological analytical manoeuvres are thus decisive to preclude the commoner entities and recognize them explicitly. Unambiguous identification of this exceedingly uncommon malignancy is decidedly critical when planning therapy, considering the differences in clinical behaviour from other commoner epithelial neoplasms. This case underscores the necessity for heightened awareness among clinicians regarding such rare tumors, as accurate and timely diagnosis is essential for optimal therapeutic strategies and improved patient outcomes.
